# Characteristics of Medial Depression of the Mandibular Ramus: A CBCT Analysis in Different Sagittal Skeletal Patterns

**DOI:** 10.30476/dentjods.2022.89659.1427

**Published:** 2023-03

**Authors:** Mahvash Hasani, Maryam Karandish, Yalda Salari

**Affiliations:** 1 Dept. of Oral and Maxillofacial Radiology, School of Dentistry, Shiraz University of Medical Sciences, Shiraz, Iran; 2 Dept. of Orthodontics, School of Dentistry, Shiraz University of Medical Sciences, Shiraz, Iran; 3 Dept. of Oral and Maxillofacial Radiology, School of Dentistry, Tehran University of Medical Sciences, Islamic Azad University, Tehran, Iran

**Keywords:** Medial depression of the mandibular ramus, Skeletal sagittal characteristics, CBCT

## Abstract

**Statement of the Problem:**

Medial depression of the mandibular ramus (MDMR) as a normal anatomical variation might complicate orthognatic surgeries that involve ramus. When planning an orthognatic surgery, it is clinically valuable to notice MDMR in osteotomy site to decrease the risk of failure.

**Purpose:**

The aim of present study was to evaluate the prevalence as well as characteristics of MDMR in three skeletal sagittal classifications.

**Materials and Method:**

This cross sectional study evaluated 530 cone beam computed tomography (CBCT) scans, of which 220 were enrolled. The skeletal sagittal classification, the presence of MDMR, the shape, depth, and width of MDMR were recorded for each patient by two examiners. Chi-square test was performed to determine the differences between three skeletal sagittal groups and between two genders.

**Results:**

The overall prevalence of MDMR was 60.45%. MDMR was mostly detected in class III (76.92%), followed by class II (76.66%), and class I (54.87%). In the analyzed CBCT scans, semi-lunar was the most common shape detected (42.85%), followed by triangular (30.82%), circular (18.04%), and tear-drop (8.27%). The depth of MDMR was not significantly different between three sagittal groups and between genders; however, the width of MDMR was higher in class III group and in male patients. In the present study, MDMR was found to be more common in patients with class II and class III skeletal classifications. Although, MDMR was more frequent in class III, the difference between class II and class III was not significant.

**Conclusion:**

More caution is needed during orthognatic surgery in patients with dentoskeletal deformities during the splitting of the ramus. Moreover, higher width of MDMR in class III and male patients should be concerned when planning an orthognatic surgery for these patients.

## Introduction

**Figure 1:**
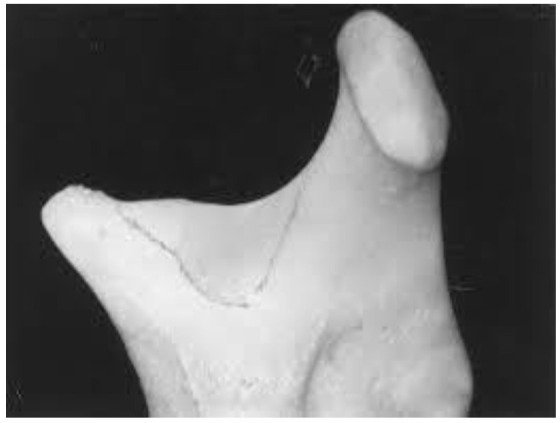
Medial depression of the mandibular ramus (MDMR) was shown by black line

Medial depression of the mandibular ramus (MDMR) or medial sigmoid depression is a normal anatomical variation first reported by Langlais *et al*. [ 1 ]. This depression is located just below and slightly anterior to the most inferior aspect of the sigmoid notch (Figure 1) [ 1 - 2 ]. This area appears as a radiolucent foramen because of a decrease in X-ray absorption and therefore, it might be misinterpreted as a pathological entity [ 3 - 4 ]. It has been reported that MDMR might complicate the splitting of ramus during orthognatic surgery due to the fusion of the medial and lateral cortical plates in patients with dentoskeletal deformities [ 5 ]. On the other hand, it is reported that this depression is associated with high muscle activity, which can increase the potential relapse in orthognatic surgery [ 6 ]. 

Ethnical and congenital factors are assumed to affect the prevalence of MDMR as reported in different ethnic population [ 7 ]. The prevalence of MDMR is ranged mostly from 5.3 to 32.7% in previous studies [ 1 , 7 - 9 ]; however, the prevalence of MDMR was reported 70% in an Indian population [ 10 ]. In addition, previous studies declared the higher prevalence of MDMR in dentoskeletal deformities using panoramic radiographs [ 7 - 11 ]. Additionally, the use of cadavers and dry skulls, concerning the difficulty in prediction of age and gender, are not at ease for studies [ 5 , 12 ].

Considering the limitations of the panoramic radiographs and dry mandibles and regarding the importance of MDMR in selection of osteotomy site with the least risk of fracture in orthognatic surgery, especially in sagittal split osteotomy and gross bone resection in mandibular corpus malignancies, the current study was designed to use CBCT scans for determining the prevalence and the characteristics of MDMR in patients with different sagittal skeletal classifications.

## Materials and Method

 A total of 530 CBCT full-face scans of patients referred to Oral and Maxillofacial Radiology Department of Shiraz Dental School were examined. Informed consent was obtained for experimentation with human subjects. The privacy rights of human subjects were observed. The study proposal was approved by Chancellor of Research, (Grant No: 13307) and Ethics Committee (IR.SUMS.REC.1396.S211) Shiraz University of Medical Sciences. A total of 310 CBCT scans were excluded considering the exclusion criteria defined as maxillofacial developmental malformation, history of previous trauma, history of previous surgical intervention in the area of the mandibular ramus, and missing permanent posterior teeth. All CBCT scans were obtained in a standardized head posture (the Frankfort plane parallel to the floor). The scans were obtained using the FDP-based CBCT (New Tom VGi, QRSrL, Italy) with following settings: 110kVp and 3.6 s exposure, and 15 cm* 15cm field of view. The CBCT scans were reconstructed 3-dimensionally so that they could be sectioned at any plane and position. The scans were divided into three skeletal sagittal classifications (class I, II, and III) according to the skeletal indices defined as Cl I (ANB: 2-5), Cl II (ANB ≥ 5), and Cl III (ANB < 2).

**Figure 2:**
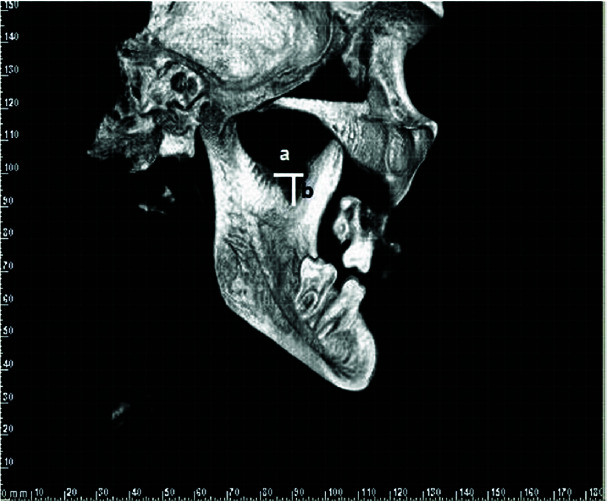
Medial depression of the mandibular ramus (MDMR) measurement: width (a), depth (b) on 3D CBCT images

**Figure 3:**
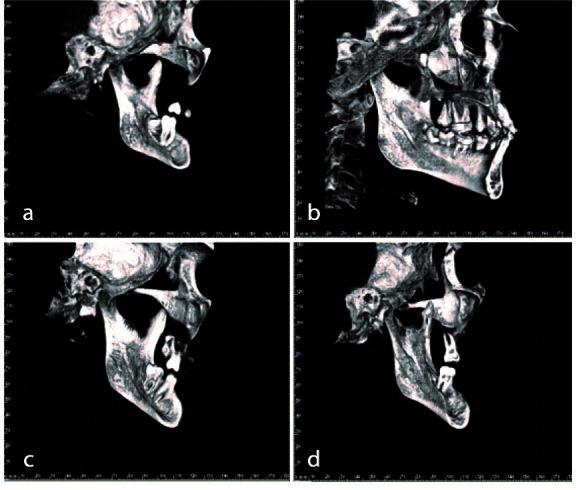
Different types of medial depression of the mandibular ramus (MDMR) based on 3D cone beam computed tomography (CBCT) images, **a:** circular shape, **b:** triangular shape, **c:** tear-drop shape, **d:** semicircular shape

As definition, A is the innermost point on the contour of premaxilla between the anterior nasal spine and the anterior tooth; B is the innermost point on the contour of the mandible between the incisor tooth and the bony chin; and N is the anterior point of the intersection between the nasal and frontal bones. In brief, ANB depicts the magnitude of discrepancy between the mandibular and maxillary jaws. The larger the ANB angle, the more convexity in facial skeletal component, leading to a class II malocclusion. Likewise, the smaller the ANB angle, the more concavity in facial skeletal component, which leads to a class III malocclusion. The presence or absence of MDMR, and if present, the shape, depth, and width of this depression were recorded (Figure 2). The geometric shapes of MDMR were defined as tear-drop, semilunar, circular, and triangular which are the types considered for interpretation in the literature (Figure 3). The depth of MDMR was identified by measuring the distance from the surface of the medial aspect of mandibular ramus to the innermost point of the depression in millimeters. To determine the width of MDMR, the distance in millimeters from the most anterior point of the anterior border of MDMR to the most posterior point of the posterior border of MDMR was measured. One oral and maxillofacial radiologist examined the CBCT scans and when a consensus was reached, the radiograph was included in the study. The relationships between the shape, the depth, and the width of MDMR and three skeletal sagittal classifications were assessed. In addition, the relationships between the shape, the depth, and the width of MDMR and the gender of the patients were evaluated. All CBCT images were analyzed with NNT software. Chi-square test was performed to analyze the results and to determine the differences between three skeletal sagittal classifications and between genders. The data were analyzed using SPSS (Statistical Package for Social Studies) version 23.00. The statistical significance was set at *p*< 0.05. 

## Results

The overall prevalence of MDMR was 60.45%. A total of 133 out of 220 CBCT scans showed MDMR (either unilateral or bilateral). MDMR was most frequently detected in class III patients (76.92%), followed by class II (76.66%), and class I patients (54.87%) (Table 1). Although MDMR was more frequent in class III, the difference between class II and class III was not statistically significant. In CBCT scans detected with MDMR, semi-lunar was the most prevalent shape identified in 42.85% of patients, followed by triangular (30.82%), circular (18.04%), and tear-drop (8.27%). The most prevalent shape in class I patients was semi-lunar, followed by triangular, circular, and tear-drop. In class II patients, both semi-lunar and circular were the most prevalent shapes, while depressions with triangular and tear-drop shapes were less common. In Class III patients, triangular presented the highest prevalence; followed by semi-lunar and circular, while, tear-drop shaped depressions were not seen in this group (Table 2). The depth of MDMR did not show a statistically significant difference in three skeletal sagittal classifications; whereas, the width of MDMR was greater in class III compared to class I and class II (*p* Value: 0.003) (Table 2). The depth of MDMR did not differ between male and female patients; the mean depth of MDMR was 5.9mm in males and 4.83mm in females. However, the mean width of MDMR in males was greater than the mean width of MDMR in females (*p* Value: 0.047) (Table 3).

**Table 1:** Prevalence of medial depression of the mandibular ramus (MDMR) in the three skeletal sagittal classifications

	Sex		Total	Patients with MDMR*	Patients with MDMR*
	Male	Female		N	%
Class I	52	112	164	90	54.87
Class II	2	28	30	23	76.66
Class III	6	20	26	20	76.92
Total	60	160	220	133	60.45

* MDMR: Medial depression of the mandibular ramus

**Table 2:** Distribution of medial depression of the mandibular ramus (MDMR) by shape predilection in three skeletal sagittal classifications

Skeletal classification	N.	Shape				Dimension			
		Semi-lunar (N.)	Circular (N.)	Triangular (N.)	Tear-drop (N.)	MDMR* depth	MDMR* width
						Mean±SD	Mean±SD
Class I	90	42	11	27	10	5.300±3.400	9.300±4.004^a^
Class II	23	9	9	4	1	4.700±1.941	7.780±3.330^b^
Class III	20	6	4	10	0	4.800±2.142	11.950±4.045^a^
Total	133	57	24	41	11	5.120±3.025	9.440±4.057
*p* Value						0.612	0.003

* MDMR: Medial depression of the mandibular ramus

**Table 3:** Comparison of medial depression of the mandibular ramus (MDMR) depth and width in both genders

	Sex	N	Mean±SD	*p* Value
MDMR* depth	Male	39	5.900±4.621	0.340
	Female	94	4.830±2.582	
MDMR* width	Male	39	11.050±3.441^a^	0.047*
	Female	94	8.980±4.561^b^	

* MDMR: Medial depression of the mandibular ramus

## Discussion

The results showed that MDMR was most frequently detected in class III (76.92%), followed by class II (76.66%), and class I (54.87%). In the analyzed CBCT scans, semi-lunar was the most prevalent shape, follow-ed by triangular, circular, and tear-drop. Although the depth of MDMR was not significantly different between three sagittal groups and between genders, the width of MDMR was higher in class III group and in male patients.

Panoramic view is a two-dimensional image and only the structures that fall within the focal trough can be trusted. In addition, the airway shadow, the pterygoid plates, the soft palate, and other structures superimposed on the sigmoid notch region might lead to misinterpretation [ 2 ]. Besides, the subjectivity of interpreting panoramic radiographs must be considered [ 7 , 10 ]. The limitations of the panoramic radiographs might be responsible for the differences between the prevalence of MDMR reported in mandibular specimens and in patients’ radiographs as well [ 7 , 9 - 10 ].

Muto *et al*. [ 12 ] and Yu *et al*. [ 5 ] have criticized the use of cadavers and dry skulls in anatomic studies because this type of material does not provide data regarding the age and the gender of the sample. Moreover, the morphology of dry skulls is usually very different from the young patients who usually undergo the correction of dentoskeletal deformities [ 11 - 12 ]. 

The prevalence of MDMR ranged from 5.3% to 32.7% in previous studies, which were mostly conducted on panoramic radiographs [ 1 - 2 , 7 - 9 ] and only one study was performed using CT scans [ 13 ]. However, in Asdullah *et al*. study [ 10 ], the prevalence of MDMR was 70% in panoramic radiographs in Indian population. In the present study, the overall prevalence of MDMR in patients’ CBCT radiographs was 60.45%. The discrepancy in the results of the current study and the previous studies might be due to the different methods used. To avoid the distortion, unequal magnification, and the superimposition of adjacent structures as the main drawbacks of using panoramic radiographs, we employed CBCT images. In addition, ethnic variability was observed among different studies. 

The results of the current study showed that MDMR was mostly detected in class III, followed by class II, and class I. In a study conducted by Carvalho *et al*. [ 7 ] who compared the prevalence of MDMR in patients with dentoskeletal deformities and class I group, higher prevalence of MDMR was found in cases with dentoskeletal deformities. Dalili *et al*. [ 8 ] and Sudhakar *et al*. [ 9 ] reports indicated that MDMR was more prevalent in class II and class III groups, although the differences reported in the present study were not statistically significant. The results of the present study and the before mentioned studies suggest examining patients to identify MDMR prior to orthognatic surgery to avoid undesirable outcomes. 

Carvalho *et al*. [ 7 ] found that triangular shape was the most prevalent one of MDMR, followed by semi-lunar, tear-drop, and circular. Sudhakar *et al*. [ 9 ] and Asdullah *et al*. [ 10 ] reported higher prevalence of semi-lunar in their studies, followed by triangular, which is similar to our study. However, in these studies, circular was the least common shape [ 9 - 10 ]. Sudhakar *et al*. [ 9 ] found higher prevalence of semi-lunar in all skeletal classifications. We found the same results in class I and class II groups; however, in our study triangular was the most prevalent shape in class III group. The differences between our results and the previous studies might be due to the different methods used. We used CBCT scans which have none of the limitations of panoramic radiography and result in a more accurate and reliable interpretation. Furthermore, the variations in the size and the shape of depression in the bone may be related to variations in muscle function. An association between MDMR and maximum bite force was observed by Adisen *et al*. [ 6 ] who noted higher values of maximum bite force in patients with MDMR. They also compared the maximum bite force between three skeletal sagittal classifications and found that the maximum bite force was higher in class I group in comparison to the other two groups, and the class III group had the lowest maximum bite force [ 6 ]. Besides, when different shapes of MDMR were compared, higher maximum bite force was reported in circular depressions, followed by semi-lunar, tear-drop, and triangular depressions [ 6 ]. This might be the reason for the higher prevalence of semi-lunar depressions in class I and class II compared to the class III and the higher prevalence of triangular depressions in class III patients in the present study.

In our study, the mean depth of MDMR was 5.12 mm and the mean width of MDMR was 9.44 mm. Kang [ 13 ] found that the mean width of MDMR was 8.3 mm in Korean population. The discrepancy in the results of the present study and Kang’s study might be explained by different ethnic characteristics and the different methods employed by two studies. Kang measured the width of MDMR on dry mandibles and we used CBCT images of the patients. In addition, the differences in the size of MDMR may be due to the variations in muscle function [ 6 ]. Because of the functional adaptation in the ramus in response to the insertion of medial and posterior attachments of temporal muscle to this area, functional patterns and bite forces play noticeable roles in determining the characteristics of MDMR [ 6 , 14 - 15 ]. We found no significant differences in MDMR depth between three skeletal sagittal classifications and between male and female patients. Our results might support the idea that there is no difference in craniomandibular muscle activity in different sagittal skeletal disharmonies.

Further studies are recommended to illuminate the exact association between MDMR characteristics and muscle function by using CBCT and other advanced modalities.

## Conclusion

MDMR was more prevalent in patients with class II and class III skeletal classifications. Although, MDMR was more frequent in class III, the difference between class II and class III was not statistically significant. Therefore, more caution should be regarded in patients with dentoskeletal deformities during the splitting of the ramus. On the other hand, the higher width of MDMR in class III and male patients should be concerned when planning an orthognatic surgery for these patients.

## Acknowledgements

This study was supported by Chancellor of Research, Shiraz University of Medical Sciences (Grant No: 13307). The authors thank Dr. M. Vosoughi from the Dental Research Development Center, for the statistical analysis.

## Conflict of Interest

The authors declare that they have no conflict of interest.

## References

[1] Langlais RP, Glass BJ, Bricker SL, Miles DA ( 1983). Medial sigmoid depression: a panoramic pseudoforamen in the upper ramus. Oral Surg Oral Med Oral Pathol.

[2] Philipsen H, Takata T, Reichart P, Sato S, Suei Y ( 2002). Lingual and buccal mandibular bone depressions: A review based on 583 cases from a world-wide literature survey, including 69 new cases from Japan. Dentomaxillofac Radi.

[3] White S, Pharaoh M (2004). Oral radiology principles and interpretation.

[4] Yeung AWK, Wong NSM ( 2021). Medial sigmoid depression of the mandibular ramus as a lesion-mimicking anatomical variation: a systematic review. Int J Environ Res Public Health.

[5] Yu IH, Wong YK ( 2008). Evaluation of mandibular anatomy related to sagittal split ramus osteotomy using 3-dimensional computed tomography scan images. Int J Oral Maxillofac Surg.

[6] Adisen M, Okkesim A, Misirlioglu M ( 2018). A possible association between medial depression of mandibular ramus and maximum bite force. Folia Morphol.

[7] Carvalho IM, Damante JH, Tallents RH, Ribeiro-Rotta RF ( 2001). An anatomical and radiographic study of medial depression of the human mandibular ramus. Dentomaxillofac Radiol.

[8] Dalili Z, Mohtavipour S ( 2003). Frequency of medial sigmoid depression in panoramic view of orthodontic patients based on facial skeletal classification. J Guilan Univ Med Sci.

[9] Sudhakar S, Naveen Kumar B, Prabhat MPV, Nalini J ( 2014). Characteristics of medial depression of the mandibular ramus in patients with orthodontic treatment needs: a panoramic radiography study. JCDR.

[10] Asdullah M, Aggarwal A, Khawja K, Khan M, Gupta J, Ratnakar K ( 2019). An anatomic and radiographic study of medial sigmoid depression in human mandible. JIAOMR.

[11] Özkan G, Sessiz AKR ( 2020). Evaluation of the frequency of medial sigmoid depression using panoramic radiographs: A retrospective study. J Dent Fac Atatürk Univ.

[12] Muto T SK, Yamamoto K, Kawakami J ( 2003). Computed tomography morphology of the mandibular ramus in prognathism: effect on the medial osteotomy of the sagittal split ramus osteotomy. J Oral Maxillofac Surg.

[13] Kang BC ( 1991). The medial sigmoid depression: its anatomic and radiographic considerations. J Korean Acad Maxillofac Radiol.

[14] Honing JF ( 1991). Identification of anatomical radiolucencies in the mandibular ramus. Electromedica.

[15] Yeung AWK, Wong NSM ( 2021). Medial sigmoid depression of the mandibular ramus as a lesion-mimicking anatomical variation: a systematic review. Int J Environ Res Public Health.

